# Detection of Breast Lesions Utilizing iBreast Exam: A Pilot Study Comparison with Clinical Breast Exam

**DOI:** 10.3390/cancers18020281

**Published:** 2026-01-16

**Authors:** Victoria L. Mango, Marta Sales, Claudia Ortiz, Jennifer Moreta, Jennifer Jimenez, Varadan Sevilimedu, T. Peter Kingham, Delia Keating

**Affiliations:** 1 Breast Imaging Service, Department of Radiology, Memorial Sloan Kettering Cancer Center, 300 East 66th Street, New York, NY 10065, USA; 2 Department of Nursing, Memorial Sloan Kettering Ralph Lauren Center for Cancer Care, 1919 Madison Avenue, New York, NY 10035, USA; 3 Department of Epidemiology and Biostatistics, Memorial Sloan Kettering Cancer Center, 1275 York Avenue, New York, NY 10065, USA; 4 Department of Surgery, Memorial Sloan Kettering Cancer Center, 1275 York Avenue, New York, NY 10065, USA; 5 Breast Imaging Service, Department of Radiology, Columbia University Medical Center, 161 Fort Washington Avenue, New York, NY 10032, USA

**Keywords:** breast cancer, mammography, iBreast Exam, clinical breast exam

## Abstract

**Background/Objectives**: The iBreast Exam (iBE) electronically palpates the breast to identify possible abnormalities. The purpose of this study was to assess iBE feasibility and compare it to Clinical Breast Exam (CBE) for breast lesion detection. **Methods**: Prospective evaluation of 300 asymptomatic women, ≥18 years old, with CBE, iBE, and mammography was performed. Sensitivity, specificity, positive predictive values (PPV), and negative predictive values (NPV) of iBE and CBE for detecting suspicious breast lesions were calculated using breast imaging as the reference standard. For women with one year follow up, the sensitivity, specificity, PPV, and NPV for cancer detection were calculated. **Results**: 300 women (mean age 58.9 years) underwent CBE, iBE, and mammography. In 2/300 (0.7%), CBE was positive; in 1/300 (0.3%), iBE was positive; and in 24/300 (8%), screening mammograms were positive. Nine had suspicious imaging findings with biopsy (three malignant and six benign). Of three cancers, all visualized mammographically, CBE and iBE detected an ipsilateral breast abnormality in one woman and missed two cancers (<2 cm). Sensitivity, specificity, NPV, and PPV of iBE and CBE were similar, with no statistically significant difference in NPV or PPV for detection of suspicious breast findings or breast cancer (*p* > 0.05). **Conclusions**: Mammography detected all breast cancers in our cohort and remains the standard of care. iBE is feasible to perform. Our pilot data demonstrates iBE performed similarly to CBE by trained nurse practitioners. Given our small study population, further investigation is warranted into the potential use of iBE where trained healthcare practitioners are not readily available.

## Simple Summary

Women without access to standard medical tests to check for breast cancer may benefit from a portable technology to check for possible cancer. This could alert a woman to seek medical care. This study compares a new portable technology designed to examine the breast, called the iBreast Exam, to standard tests including clinical breast exam (a breast examination performed by a nurse with their hands) and an X-ray picture of the breast (mammography). In this pilot study of 300 women, mammography found three breast cancers while iBreast Exam and clinical breast exam found one cancer. Cancers missed by the iBreast Exam and clinical breast exam were small (<2 cm). Mammography remains the best way to check for breast cancer. Given the iBreast Exam performed similarly to the clinical breast exam, it may be helpful in areas where trained healthcare workers are not available to provide a clinical breast exam. Further research is needed.

## 1. Introduction

Breast cancer screening with mammography reduces mortality and is the standard of care for early breast cancer detection [1,2,3,4]. However, there are disparities in screening mammography use, particularly for black and Hispanic populations, in the United States [5]. People living in areas at greater socioeconomic disadvantage are less likely to have accredited breast imaging facilities nearby, contributing to disparities in breast imaging access [6].

Imaging access disparities are even greater in low- and middle- income countries (LMIC), where breast cancer incidence and mortality is rising. In LMIC, breast imaging is a limited resource, and large population-based breast cancer screening with mammography is not possible given financial, logistical, and resource constraints [7,8]. In such settings, breast cancer detection often relies on clinical breast examination (CBE) to determine which patients should undergo further evaluation [8,9,10]. While CBE can downstage breast cancer at diagnosis and reduce mortality [11], reported sensitivities range widely from 22 to 85% [12,13,14].

**Figure 1:**
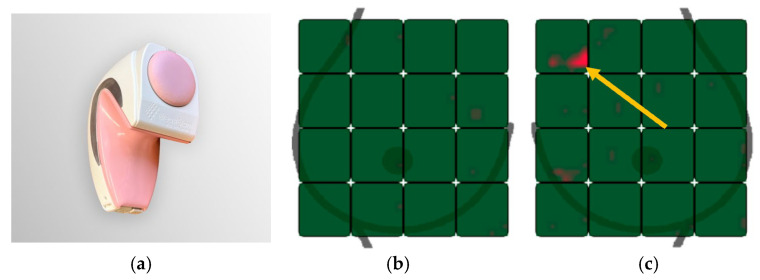
(**a**) iBE device; (**b**) iBE color map output demonstrating a normal breast exam displaying all green; and (**c**) an abnormal exam with a bright red focus (arrow) in the upper inner breast, indicating an area needing further evaluation.

Given CBE limitations and limited imaging access, women in low resource settings could potentially benefit from a low-cost accessible alternative for breast cancer detection. The iBreast Exam (iBE) (UE Life Sciences, Philadelphia, PA, USA) is a 510(k) FDA-cleared, portable, battery powered device designed for use with minimal training to assess the breast for areas that warrant further evaluation (shown in Figure 1) [15,16]. The iBE does not utilize radiation. It does not distinguish benign from malignant lesions, but identifies a possible breast abnormality that should undergo breast imaging and/or clinical evaluation. The iBE is a painless exam utilizing dynamic capacitive pressure sensors to perform electronic breast assessment and measure tissue elasticity through capacitive measurements enabling detection of tissue stiffness variations when pressed against the skin [16]. The device detects induced voltage from tissue displacement which mimics palpation [16,17,18,19]. Variations in tissue displacement and stiffness are detected if there is a mass within the breast. The device is not designed to detect other findings, such as microcalcifications, which are not expected to be palpable. Focusing on screening for palpable breast masses alone may still enable the identification of clinically relevant breast cancer diagnosed at a lower stage at presentation [11]. Wirelessly transmitted results from the iBE display on a tablet and can be sent to a cloud. The iBE produces a color map with a red focus indicating an area needing additional evaluation.

Initial iBE studies in the United States and India showed high negative predictive values (NPV) with sensitivities of 84–87% and specificities of 80–94% [17,18,19,20,21,22]. More recently, we studied 424 Nigerian women who had iBE performed by nurses demonstrating a high sensitivity and NPV, but lower specificity than a surgeon’s CBE in identifying suspicious breast lesions [23], supporting a need for further study. A 2023 systemic review by Bhimani et al. of 11 iBE studies noted a wider sensitivity range of 34.3–86% and specificity range of 59–94% as well as a high false positive rate [24]. In addition, the authors note that the heterogeneity of available iBE studies limits the ability to conduct a meta-analysis or quantitative synthesis of iBE performance [24]. Valdez et al. examined a small patient population in Guam (39 women) and reported an even lower sensitivity, 20%, with a specificity of 92% [25]. Additionally, a 2024 study from Portugal by Goncalves et al. utilized an iBE prototype and highlighted the need for specific device technical improvements to improve performance [26]. Such wide variations in reported sensitivities and specificities in the literature, the heterogeneity of previously published studies, and the need for device technical improvements support the need for further study with standardized imaging protocols for comparison.

Since our study in Nigeria, the iBE device has undergone modifications to improve specificity, which warrants a study with blinded comparison to CBE and consistent imaging protocols. While both iBE and CBE are intended for clinical use, they vary in the operator skill and the training required to perform the exam, which is much greater for CBE. The iBE does not require a skilled operator to perform the exam or a physician to interpret the results [24]. Furthermore, point-of-care technologies, such as the iBE, offer the opportunity to decentralize cancer diagnosis with the potential, if validated, to provide quick, affordable, and scalable cancer screening in resource-limited environments to reduce cancer disparities [27,28,29].

The Memorial Sloan Kettering (MSK) Ralph Lauren Center (RLC) provides cancer diagnosis, treatment, and support services in the Harlem community of New York City [30]. Women present for same day CBE by a Nurse Practitioner (NP) and screening mammography by a radiology technologist (RT). The objective of this pilot study was to assess the feasibility of performing iBE and compare its performance to mammography and CBE for suspicious breast lesion detection in women presenting for mammography screening. This study is motivated by the need for a breast examination device that can be used in low resource settings, by individuals with limited training, to identify women in need of further breast evaluation.

## 2. Materials and Methods

### 2.1. Overall Study Design

This prospective, Health Insurance Portability and Accountability Act compliant study was IRB approved by the MSK IRB (protocol 19-302, NCT 04761055). Women, 18 years or older, scheduled for screening mammography and CBE at the RLC, were recruited, and written informed consent was obtained. Participants provided demographic information and history and underwent three examinations on the same day: CBE, iBE, and mammography. A RedCap database was used to record results. The small study population size was dictated by funding constraints, and thus this was deemed an initial pilot study.

### 2.2. Clinical Breast Exam

Participants first underwent CBE by one of two NPs with 25 and 15 years clinical experience. Women were systematically examined by breast palpation while positioned upright and supine. Abnormal findings were recorded as positive and a normal exam negative.

### 2.3. iBreast Exam

After CBE, participants underwent iBE (UE Life Sciences, Inc., Philadelphia, PA, USA) by one of two RTs who were blind to CBE results. Women were supine with the device systematically placed on the breast. A red focus on the color map was considered positive and green negative.

RTs had no prior iBE experience. They underwent virtual training, and prior to study enrollment, each performed 10 iBEs monitored by the study PI to establish technical proficiency. At the end of the study, RTs were surveyed regarding their experiences with the iBE.

### 2.4. Breast Imaging

After iBE, standard mammogram (Pristina Mammography System, GE HealthCare, Chicago, IL, USA) was performed and interpreted by one of 17 radiologists, all with breast imaging fellowship training or >25 years breast imaging experience. Radiologists were not blind to CBE/iBE results. This is because in our standard clinical practice, an area of concern within the breast on exam is marked on the skin with a radiopaque marker, so any radiologist looking at the image can clearly identify on the image if there is an area of specific concern. In order to maintain our standard clinical care, the patients in this study were managed the same. Screening mammograms with a BI-RADS 0 assessment were positive and those with a BI-RADS 1 or 2 assessment were negative. This follows the American College of Radiology (ACR) guidelines for classifying screening mammogram results [31].

Women with positive CBE, iBE, and/or screening mammogram findings underwent diagnostic imaging with additional mammographic views and/or ultrasound (US) to determine if a breast lesion was present. Positive iBE findings were imaged the same as a positive CBE, with a targeted breast US and, if needed, additional mammographic views.

If there was a lesion on diagnostic imaging, management was determined by the radiologist (i.e., biopsy, short interval follow up, or routine screening). Positive iBE or CBE findings without an imaging correlation were managed clinically. Diagnostic imaging with a BI-RADS 4 or 5 assessment, indicating a breast biopsy was recommended, was considered positive for a suspicious breast lesion. Management was based on biopsy results. Pathology results were recorded and correlated with CBE, iBE, and imaging findings. Breast density was based on the radiologist’s mammogram report [31].

### 2.5. Statistical Analysis

iBE and CBE performance for detecting suspicious breast lesions was evaluated using imaging findings as the reference standard for all individuals. For individuals that had biopsy or long-term follow-up, the corresponding findings were used as the gold standard for the presence or absence of cancer. Sensitivity and specificity were estimated and compared between iBE and CBE using McNemar’s test. PPV and NPV were estimated and compared between iBE and CBE using Leisenring’s test. All analyses given here are considered exploratory, and therefore ongoing research is required to validate the findings. All analyses were conducted using R 4.2 software. Type I error rate for all statistical tests was set to 0.05 (α). Kappa statistics were also estimated to quantify the agreement between iBE/CBE and the outcomes. Given the small sample size of the studied population, which was dictated by funding constraints, the statistical power is greatly reduced for determining sensitivity and performing subgroup analyses.

## 3. Results

### 3.1. Patient Population

A total of 300 asymptomatic women presenting for screening mammography were prospectively enrolled, with a mean age of 58.9 years (range 29–83 years). Self-reported race included: 151/300 (50.3%) black, 81/300 (27.0%) white, 9/300 (3.0%) Asian, and 59/300 (19.7%) other races. A total of 125/300 (41.7%) reported Hispanic ethnicity, and 109/300 (36.3%) had a family history of breast or ovarian cancer. None reported a known genetic mutation. A total of 9/300 (3.0%) presented for baseline screening mammography, and 291/300 (97.0%) previously had a mammogram.

All 300 women underwent CBE, iBE, and screening mammography and were included in the analysis.

### 3.2. Breast Exam Results

**Figure 2:**
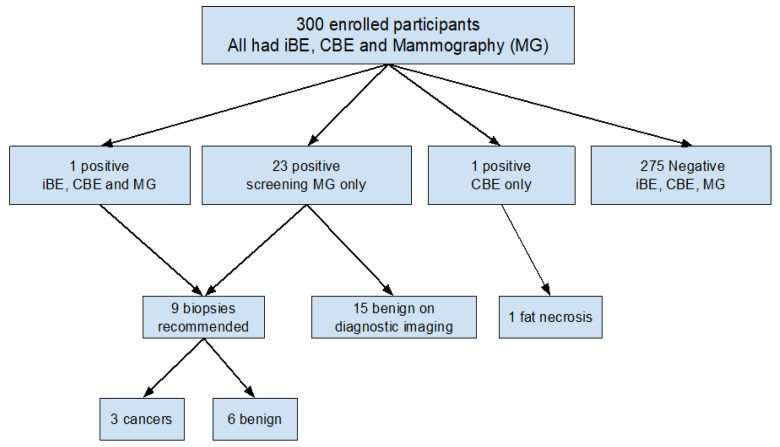
Results of the studied population undergoing breast evaluation (iBE = iBreast Exam, CBE = Clinical Breast Exam, and MG = Mammography).

Of 300 women, 2/300 (0.7%) had a positive CBE and 1/300 (0.3%) had a positive iBE (shown in Figure 2). Initial screening mammograms were positive (BI-RADS 0) in 24/300 (8.0%) of women. 142/300 (47.3%) of women had dense breasts.

Of 25 women recommended for additional diagnostic breast imaging (23 positive on screening mammogram alone, 1 positive on CBE alone, and 1 positive on all three exams), 9 had suspicious findings (BI-RADS 4 or 5) on diagnostic breast imaging with a breast biopsy recommended (shown in Table 1).

CBE and iBE were compared for the detection of suspicious breast lesions (BI-RADS 4 or 5) using breast imaging as the reference standard. Sensitivity, specificity, PPV, and NPV for detecting BI-RADS 4 or 5 breast lesions for CBE vs. iBE are summarized in Table 2. For the subset of women who had at least one year follow up (*n* = 236), the sensitivity, specificity, PPV, and NPV for breast cancer were calculated. Note that both the sensitivity and specificity have wide confidence intervals, likely due to the small sample size of the study population. *p* value calculations for sensitivity and specificity are limited in this feasibility study given the small number of disagreements found between the iBE and CBE.

Confusion matrices were generated and Kappa statistics were estimated to quantify agreement between iBE/CBE and outcomes as shown in Table 3. Agreement ranges from moderate to poor.

### 3.3. Biopsy Recommendations and Pathology Results

Nine breast biopsies were performed yielding six benign results and three malignancies: two invasive ductal carcinomas (IDC), with 1.9 cm and 1.2 cm masses, and one ductal carcinoma in situ (DCIS), with 1.2 cm calcifications. Of the three cancers, CBE and iBE detected an ipsilateral breast abnormality in 1/3 (33.3%) and missed two, a 1.9 cm IDC and 1.2 cm DCIS. All three cancers were visualized with mammography. The overall cancer detection rate (CDR) was 3/300 (1%) or 10 per 1000.

**Figure 3:**
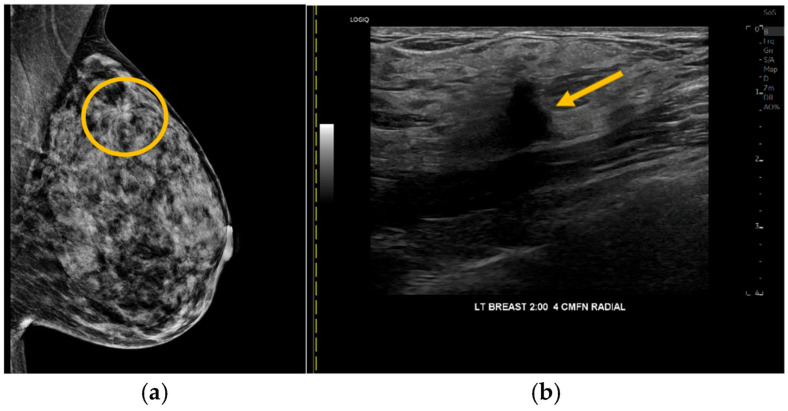
Breast cancer with positive CBE, iBE, and imaging. (**a**) Mammography demonstrates in the left upper outer breast, posterior depth, a partially obscured mass (circle) with associated architectural distortion. (**b**) Ultrasound demonstrates a correlating irregular 1.2 cm hypoechoic mass (arrow) biopsied under ultrasound guidance yielding invasive ductal carcinoma.

A single patient with a positive iBE, CBE, and mammogram was diagnosed with a 1.2 cm IDC in the upper outer left breast (shown in Figure 3). While CBE was positive in the upper outer breast, iBE was positive in the upper inner breast. The participant with positive CBE only had fat necrosis on imaging. One IDC was seen with mammography and US but had a negative CBE and iBE.

236/300 (78.7%) women had at least one year imaging follow up. No additional cancers were diagnosed in this subgroup. Sensitivity, specificity, PPV, and NPV for detecting breast cancer for CBE vs. iBE was calculated for this group and is summarized in Table 2.

### 3.4. Operator Experience with iBE

RTs performing iBE were surveyed regarding their experience with the device. Both found it easy to use (rated 9 or 10 out of 10). They both reported occasional technical difficulties including temporary wireless connection failure between the iBE and the tablet, either initially or during an exam. They both found it easy to read the iBE output (both 9 out of 10). Both RTs reported patients were physically comfortable during the exam and estimate that each iBE took ~10 min.

Both RTs mentioned challenges related to patient body habitus. With slim patients, they noted that at times, the device would initially detect ribs or the sternum as red on the iBE output, although this was clear while scanning and confirmed artifactual with patient repositioning. Currently, for larger breasts, one display tile represents a large scanning area. The RTs suggested either more tiles and/or multiple sensor sizes to accommodate different sized breasts. Currently, to accommodate larger breasts, the same tile would be utilized to scan more than one area, which could contribute to inaccurate location assignment of a positive finding. Notably, technical difficulties related to wireless connection failure and patient body habitus did not interfere with the ultimate successful completion of iBE in our study population.

## 4. Discussion

Our study of a novel, highly portable, hand-held breast examination device, the iBE, demonstrates iBE feasibility in a potentially underserved community in New York City with comparable iBE and CBE performance, both of which are inferior to mammography. The transferability of these research findings from a facility affiliated with a high-resource academic center to a clinical setting in a low resource environment is unknown. Our previous study in Nigeria and additional studies from India and Guam indicate the device is well accepted by patients and feasible for use globally [20,23,24,25].

We found training to perform iBE is minimal, as supported by prior studies [24]. Mammography RTs were trained to perform iBE through one virtual teaching session and 10 initial exams, much less than required to establish CBE proficiency. While both iBE and CBE are intended for clinical examination of the breast, they vary considerably in terms of the required operator skill and experience, with greater training and experience requirements for performing CBE. Notably, the iBE output does not require expertise for interpretation, limiting human subjectivity, and provides real-time results. This supports the potential for this device to be used in low resource settings by individuals with minimal training.

RTs found iBE easy to perform and women found it a physically comfortable exam. The iBE was performed in ~10 min, comparable to our previously published results [23]. RTs noted occasional challenges with the device’s wireless connection and in scanning women of varied breast sizes. While our study did not quantify the number and extent of the technical challenges faced, we do not have reason to believe these challenges compromised the diagnostic accuracy of the device. All iBE were successfully completed. We intend to build on this initial feedback and quantify technical challenges in future studies.

Our study demonstrated a high CDR of screening mammography, 10 per 1000, more than the expected 3–4 per 1000 [32]. Of the three cancers diagnosed, iBE and CBE were positive in the ipsilateral breast in only one, a 1.2 cm IDC. Slight location discrepancies of the iBE and CBE findings in this case may reflect iBE detection of adjacent tissue changes in the vicinity of the cancer, indicate breast mobility between exams as patients are positioned differently between CBE and iBE, or highlight that the iBE pressure map (shown in Figure 1b,c) is an approximation and not an exact indication of the location of a finding within the breast. This is an important direction for future study. The second IDC was detected with imaging alone, located deep within the breast on US, which likely contributed to challenges palpating this finding. The third cancer, 1.2 cm microcalcifications without a mass, would not be expected to be palpable with CBE or iBE. Given 97% of our population previously had mammography, the size of cancers detected in our population is expected to be small, and therefore they are less likely to be palpable. The sensitivities and specificities demonstrated a wide confidence interval, likely due to our small sample size, which contributes to data uncertainty and supports further study with a larger patient population. Allowing for this, both CBE and iBE sensitivities are lower for smaller lesions (≤2 cm) compared to larger lesions (>2 cm) [23]. Similarly, previous studies reported that cancers missed by iBE were small (<1.0 cm) [21,22]. This is important to note, as patients should not be falsely reassured by a negative iBE result given a small, highly treatable cancer may be missed.

In our small study, iBE showed no false positives (specificity 100%), while CBE had one false positive due to fat necrosis. This shows improved iBE specificity compared with an earlier device version utilized in our previous study, which showed a specificity of 50%. Given the intended use of the iBE, in a low resource setting to determine which women should undergo further evaluation, too many false positives may consume valuable resources to evaluate women who ultimately are found not to have breast cancer.

Mammography remains the standard of care for breast cancer screening, supported by our study results demonstrating the superior CDR of mammography. Given iBE performed by RTs with minimal training performed similarly to CBE by an experienced NP, it is possible future study may support iBE use in a resource-limited setting where healthcare workers trained in CBE are not available. While our study provides useful feasibility data, further research with larger multicenter trials with more cancers and blinded radiologist imaging assessments are needed prior to clinical adoption. Given our small study population, definitive conclusions about applications in a real-world clinical environment and contexts for iBE deployment, for example, in the hands of community health workers in rural settings, necessitate further study. Our group is currently undertaking an iBE study in Nigeria with community health nurses, who provide an essential healthcare resource for cancer care in Africa [33,34]. Furthermore, a cost–benefit analysis would also be prudent to evaluate the iBE in both low- and high-resource settings.

Study limitations include the small study sample, which was necessary due to funding constraints. This greatly reduces the statistical power for determining sensitivity and performing subgroup analyses. Given the exploratory nature of this study, *p* values must be interpreted with caution. The small number of disagreements between iBE and CBE limits *p* value comparisons for sensitivity and specificity. The very small number of cancers identified (*n* = 3) limits the reliability of the calculated sensitivities for both iBE and CBE. Categorizing screening mammograms with BI-RADS 0 results as positive, while standard per ACR criteria, impacts the rate of false positive mammography results in our cohort. Most of our cohort previously had mammography, which likely contributed to the small size of detected cancers and biased our detection of smaller lesions, which are less likely to be palpable. Our study did not examine inter-operator variability of the iBE, which is an important area for future research, given the device is intended to be used by individuals with limited training. Additionally, the radiologists were not blind to CBE or iBE results, which may have influenced their mammographic interpretation, which served as the reference standard in this study. Our population has access to breast imaging at a facility that is part of a high-resource large academic center, and are therefore unlikely to rely on iBE. The transferability of these results to a low-resource setting is not known and is an important area of future study. We did not have follow up on all patients, limiting our analysis of cancer detection to a subset of patients.

## 5. Conclusions

In conclusion, performing iBE is feasible, easy to learn, and well-tolerated by patients. For women with access to screening mammography, imaging remains the standard of care. Our pilot data demonstrates the iBE performed similarly to CBE by trained NPs. In settings where iBE and CBE perform similarly, CBE would be preferred. Further study is warranted into the potential use of iBE in settings where trained healthcare practitioners are not readily available to perform CBE.

In summary, the benefits of iBE include device portability, availability in low resource settings, simplicity of training, and real-time results that do not require user interpretation. The disadvantages include wide reported sensitivity and specificity results, limited detection for small cancers, and that women with a positive result must still undergo breast evaluation, given the device does not distinguish between benign and malignant lesions.

Future directions include larger multi-institutional studies with radiologists blinded to iBE/CBE results, as well as a cost–benefit analysis to determine the most appropriate clinical use of iBE.

## Figures and Tables

**Table 1:** Women recommended for breast biopsy (*n* = 9) yielding 3 malignant results and 6 benign results.

	Imaging Finding	iBE Results	CBE Results	Biopsy Results
Malignant	mass	positive	positive	IDC ^1^
	mass	negative	negative	IDC
	calcifications	negative	negative	DCIS ^2^
Benign	focal asymmetry	negative	negative	PASH ^3^
	mass	negative	negative	Angiolipoma
	architectural distortion	negative	negative	Stromal fibrosis
	architectural distortion	negative	negative	Radial scar
	mass with calcifications	negative	negative	Fibroadenoma
	mass	negative	negative	PASH

^1^ IDC = invasive ductal carcinoma. ^2^ DCIS = Ductal Carcinoma in Situ. ^3^ PASH = Pseudoangiomatous Stromal Hyperplasia.

**Table 2:** iBE and CBE sensitivity, specificity, negative predictive value (NPV), and positive predictive value (PPV) for suspicious breast imaging findings (BI-RADS 4 or 5) (*n* = 300 women) and for breast cancer (*n* = 236 women, the subset of the study population who had at least one year follow up).

	Sensitivity ^1^ (95% CI)	Specificity ^1^ (95% CI)	NPV (95% CI)	PPV (95%CI)
BI-RADS 4 or 5 Imaging Finding (*n* = 300)				
iBE	0.111 (0.003–0.482)	1.000 (0.987–1.000)	0.973 (0.948–0.988)	1.000 (0.025–1.000)
CBE	0.111 (0.003–0.482)	0.997 (0.981–1.000)	0.973 (0.948–0.988)	0.500 (0.013–0.987)
			*p* = 0.42	*p* = 0.18
Breast Cancer (*n* = 236)				
iBE	0.333 (0.008–0.906)	1.000 (0.984–1.000)	0.991 (0.970–0.999)	1.000 (0.025–1.000)
CBE	0.333 (0.008–0.906)	0.996 (0.976–1.000)	0.991 (0.970–0.999)	0.500 (0.0126–0.987)
			*p* = 0.99	*p* = 0.18

^1^* p* values for sensitivity and specificity differences were not estimable due to an insufficient number of disagreements between the two modalities.

**Table 3:** (**a**) Confusion matrices demonstrating iBE and CBE results (positive or negative) relative to positive or negative final breast imaging results (*n* = 300 women). (**b**) Demonstrated iBE and CBE results relative to the presence or absence of breast cancer (*n* = 236 women, the subset of the study population who had at least one year follow up).

(**a**)			
	**Imaging** **Negative**	**Imaging** **Positive**	**Kappa** **Value**
iBE negative	291	8	
iBE positive	0	1	0.195
			
CBE negative	290	8	0.173
CBE positive	1	1	
(**b**)			
	**Breast Cancer** **Negative**	**Breast Cancer** **Positive**	**Kappa** **Value**
iBE negative	233	2	
iBE positive	0	1	0.497
			
CBE negative	232	2	
CBE positive	1	1	0.394

## Data Availability

Anonymized participant data can be made available upon request to the corresponding author between 9 months and 36 months after publication. Proposals will be reviewed on the basis of scientific merit. After approval of a proposal, data can be shared via a secure online platform after signing a data access agreement.

## Abbreviations

The following abbreviations are used in this manuscript:

iBE	iBreast Exam
CBE	Clinical Breast Exam
NPV	Negative Predictive Value
PPV	Positive Predictive Value
NP	Nurse Practitioner
LMIC	Low- and Middle-Income Countries
MSK	Memorial Sloan Kettering
RLC	Ralph Lauren Center
RT	Radiology Technologist
MG	Mammography
IDC	Invasive Ductal Carcinoma
DCIS	Ductal Carcinoma in Situ
PASH	Pseudoangiomatous Stromal Hyperplasia
